# Social support, gender and patient delay

**DOI:** 10.1038/bjc.2011.87

**Published:** 2011-04-12

**Authors:** A F Pedersen, F Olesen, R P Hansen, R Zachariae, P Vedsted

**Affiliations:** 1 The Research Unit for General Practice, Aarhus University, Bartholins Allé 2, Aarhus DK-8000, Denmark; 2 The Danish Cancer Society and the Novo Nordisk Foundation Research Centre for Cancer Diagnosis in Primary Care (CaP), Aarhus University, Aarhus, Denmark; 3 Psychooncology Research Unit, Department of Psychology, Aarhus University, Aarhus, Denmark

**Keywords:** marital status, partner avoidance, partner support, patient delay, social support

## Abstract

BACKGROUND: The purpose of this study was to examine the relationship between perceived social support and patient delay (PD) among female and male cancer patients.

METHODS: A population-based study with register-sampled cancer patients was designed. Patient delay was defined as the time interval between the patient's experience of the first symptom and the first contact with a health-care professional. Both dates were provided by the patients (*n*=910). The patients completed a purpose-designed questionnaire, which assessed the patient's perceptions of how the partner reacted (‘Partner Avoidance’ and ‘Partner Support’) and how others in the social network responded (‘Other Avoidance’ and ‘Other Support’) to the patient's worries about the symptoms. The associations between the social support subscales and PD were analysed separately for men and women.

RESULTS: In female patients, Partner Support and Other Support were associated with shorter PD, whereas Other Avoidance was associated with longer PD. In the multivariate analysis, Other Avoidance remained associated with longer PD. Moreover, disclosure of symptoms to someone reduced the likelihood of a long PD in female patients. In male patients, none of the social support scales significantly increased or decreased the risk of a long PD in the univariate analysis, but Partner Support significantly decreased risk of a long PD in the multivariate analysis.

CONCLUSIONS: The results of this study suggest that social support and avoidance from network members influence length of PD differently in male and female cancer patients. This gender difference may explain previous mixed findings obtained in this field.

Several studies have revealed associations between cancer mortality and indicators of social support such as perceived social support, marital status and network size (Pinquart and Duberstein, 2009). The causal pathway is uncertain, but health behaviour may be a mechanism that mediates the association between social support and cancer mortality. For instance, members of the social network may motivate cancer patients to implement a healthy diet and encourage patients to avoid health-damaging behaviour such as cigarette smoking and alcohol consumption (Pinquart and Duberstein, 2009). The results of literature reviews suggest that approximately 20–30% of cancer patients delay seeking help for more than 3 months after having experienced possible symptoms of cancer (Richards *et al*, 1999a; Tromp *et al*, 2005; Scott *et al*, 2008). As delay in cancer diagnosis and treatment is an important factor for prognosis (Richards *et al*, 1999b), it is natural to ask whether members of the social network may stimulate an individual who experiences an unexplained symptom to seek medical help, and whether people with a well-established network are diagnosed earlier and hence have a better prognosis than people with a constrained social network.

On the basis of the existing literature, it is not possible to draw a definitive conclusion regarding the influence of social support on patient delay (PD). One systematic review of delayed presentation of breast cancer symptoms revealed no effect of marital status on PD, but revealed that women who disclosed their discovery of a symptom within a week of discovery were less likely to delay help seeking (Ramirez *et al*, 1999). In a systematic review of PD in the diagnosis of colorectal cancer, social networks and support were identified as potentially important factors in reducing delay when patients either sought advice from or made decisions based on the experience of others (Mitchell *et al*, 2008). The level of evidence was designated as moderate, that is, <75%, but more than or equal to 50% of the studies considering the influence of social support on PD provided evidence of such a relationship. A paper that summarised the results of studies identified through the literature searches to the above-mentioned two systematic reviews reported that the influence of social support on PD appeared to vary by cancer type (Macleod *et al*, 2009). For instance, lower levels of social support were found to be associated with increased delay in women with endometrial cancer, but unrelated to delay for lung, upper gastrointestinal and urological cancers.

The mixed findings concerning the influence of social support on PD may be explained by a number of factors. First, the existing studies are heterogeneous with respect to operationalising social support. Second, there is considerable variation in the cut-offs used to designate long *vs* short PDs, and there is no gold standard concerning when is the right time to seek medical help for a symptom. Whereas a long PD may be important for cancer prognosis, a very short PD may be a consequence of somatosensory amplification, that is, the tendency to experience somatic sensations as noxious and highly disturbing (Barsky, 1992), and hypochondriacal beliefs and behaviour (Duddu *et al*, 2006). A third factor responsible for the mixed findings may be that none of the studies have addressed whether the influence of social support provided by different sources varies by gender. This may be relevant as previous studies have documented that men and women benefit differently from the support provided by social network members. For instance, the results of one study of a randomly selected community sample of men and women showed that women had larger networks of support and were more likely to rely on friends and children for emotional support, whereas men were more likely to rely exclusively on their partners (Antonucci and Akiyama, 1987). In a study of gender, marital status and the social control of health behaviour, it was seen that marriage was associated with receipt of considerably more efforts to control health for men than for women (Umberson, 1992). In addition, married men were more likely to identify their spouse as the one who tried to influence their health than married women. Fourth, the vast majority of studies examining the association between social support and PD have focused on the benefits of social support. However, one's social network is not always helpful in times of crisis. Behaviour intended to be helpful may be perceived as unsupportive by the patient and may thus have negative consequences. Such behaviour could be uninvited advice, generalising the unique experiences of the patient, enforced cheerfulness and avoidance (Zakowski *et al*, 2003; Herzer *et al*, 2006). Thus, the benefits of talking with others depend on their responses, and to our knowledge, no studies have addressed the influence of negative social support on PD to date.

In the light of this, the objective of this study was to examine the association between PD in cancer diagnosis and perceived social support provided by the partner and other members of the social network. Unlike previous studies, we explored whether the association between social support and PD varied between female and male patients, and whether perceived unsupportive behaviour from the partner or other members of the network influenced PD.

## Materials and Methods

We performed a population-based study set in the Aarhus County, Denmark, which has 640 000 inhabitants and ∼3000 new cancer cases diagnosed per year. More than 98% of the Danish citizens are registered with a general practitioner (GP), who carries out initial diagnostic investigations and refers patients to hospitals or outpatient clinics when relevant.

The study population included all incident cancer patients during the 1-year period from 1 September 2004 to 31 August 2005. An incident cancer was defined as a new cancer diagnosis excluding recurrent cancers of the same type. Thus, patients could have had another cancer type earlier. Patients younger than 18 years and patients with non-melanoma skin cancers were excluded. Patients were identified from the County Hospital Discharge Registry (HDR), which for each hospital admission and outpatient visit holds the patient's unique civil registry number (CRN), dates of admission and discharge and diagnoses classified according to the International Classification of Diseases (ICD-10).

### Data collection

We linked the patient's CRN to the County HSR in order to identify the patient's GP. The patient's GP was sent a questionnaire asking the GP to confirm the diagnosis. Patients with a confirmed diagnosis were sent a questionnaire, and non-responders received a reminder after 3 weeks.

### Patient delay

Patient delay was defined as the time interval between the date when the patient experienced the first symptom and the date when the patient made their first appointment with a health-care professional. Both dates were provided by the patient. As length of PD tend to be non-normally distributed, PD was classified as ‘short’, ‘medium’ or ‘long’ on the basis of 25th and 75th percentiles: short PD, ⩽25th percentile; medium PD, >25th to ⩽75th percentile; and long PD, >75th percentile.

### Social support

For the purpose of this study, a social support questionnaire consisting of 20 items was developed (see Appendix). The questionnaire assessed the patient's perceptions of how his/her partner and how others in the social network reacted to the worries associated with experiencing a symptom. ‘Others’ in the social network were defined to the participants as ‘children, other family members, friends, colleagues and so on’. On the basis of the explorative factor analysis, four subscales of the questionnaire were extracted: ‘Partner Avoidance’, ‘Partner Support’, ‘Other Avoidance’ and ‘Other Support’ (Hansen, 2008). Each subscale consisted of five items, and each item was scored on a 4-point Likert scale ranging from 0 (‘not at all’) to 3 (‘very much’). A mean item score was constructed for each subscale, and the minimum and maximum scores of each subscale were 0 and 3, respectively. Examples of items from the Partner Avoidance subscale are ‘My partner minimised my concerns’ and ‘My partner avoided talking about cancer’, and a high score reflected high Partner Avoidance. Examples of items from the Partner Support subscale are ‘My partner asked about my symptoms’ and ‘My partner advised me to talk to my physician’, and a high score reflected high Partner Support. The Partner Avoidance and Partner Support subscales were only completed by patients with a partner. Examples of items from the Other Avoidance subscale are ‘Others minimised my concerns’ and ‘Others were not worried’, and a high score reflected high Other Avoidance. Examples of items from the Other Support subscale are ‘Others took the initiative to talk about my concerns’ and ‘Others advised me to talk to my physician’, and a high score reflected high Other Support. In this study, the Partner Avoidance subscale had a Cronbach's *α*=0.7. All other subscales had a Cronbach's alpha of ⩾0.8.

### Covariates

For each patient, information about age, gender and cancer diagnosis was obtained from the HDR. Information on educational level, relationship status, family history of cancer and disclosure of symptoms was provided by the patient. Education was categorised into three levels: (1) lower secondary or none (UNESCO level 0–2), (2) upper secondary (UNESCO level 3–4) and (3) tertiary (UNESCO level 5–6) (UNESCO, 1997). Regarding relationship status, patients reported whether they had a partner or perceived themselves to be single at the time of diagnosis. The family history of cancer was assessed asking the patient to consider the following statement: ‘Many of my relatives have had a cancer disease’. If the patient responded ‘true’ or ‘mainly true’, they were classified as having ‘many relatives with cancer’, and if the response was ‘false’ or ‘mainly false’, they were classified as having ‘few relatives with cancer’. Patients with a partner were asked whether they had disclosed their symptoms to their partners and/or to other individuals in the social network. Single patients were asked to report whether they had disclosed their symptoms to someone in the social network. On the basis of this information, a disclosure variable was constructed reflecting whether the patient had or had not disclosed his/her symptoms to someone.

### Analysis

Data on age, gender and cancer diagnosis were obtained for both questionnaire responders and non-responders. Differences between responders and non-responders were explored with *χ*^2^ and Student's *t*-tests for independent variables. A mean item score for each of the four social support subscales was calculated for patients with a maximum of one missing item per subscale. Univariate and multivariate multinomial logistic regression analyses were used to examine associations between social support subscales, covariates and the three categories of PD (short, medium and long). Patients with a medium PD were compared with patients with a short PD and with patients with a long PD. The multivariate multinomial logistic regression analysis only included patients in a relationship, as the Partner Avoidance and Partner Support subscales were only completed by this group of patients. The following covariates were entered into the multivariate analysis: age, educational level (lower secondary or none *vs* upper secondary and tertiary combined, and tertiary *vs* upper secondary and lower secondary or none combined) and family history of cancer. The multinomial logistic regression analyses were conducted separately for male and female cancer patients. Statistical significance was determined at a *P*-value less than or equal to 0.05.

## Results

The questionnaires were completed by 1252 (53%) of the 2356 patients with a confirmed cancer diagnosis. An analysis of non-responders revealed no differences between respondents and non-respondents with regard to age, gender or diagnoses (data not shown). Of the 1252 questionnaire responses, 260 had to be excluded as the patients did not provide sufficient data for calculation of PD. Another 82 patients were excluded as they had more than one missing item per social support subscale. Hence, data on 910 cancer patients were included in the analyses. Excluded patients (mean=67.6, s.d.=13.1) were significantly older than included patients (mean=61.8, s.d.=14.0; *t*=6.63, *P*<0.001), but no gender differences were revealed between excluded and included patients (*χ*^2^=0.42, *P*=0.516).

Sociodemographic characteristics of female and male patients are shown in Table 1 . Male cancer patients were older than female patients, and more male than female cancer patients were in a relationship.

It should be noted that male patients reported more Partner Support than female patients, and that female patients reported more Partner Avoidance and Other Support than men. No difference in Other Avoidance between female and male patients was observed.

No difference was found between female and male patients with regard to the number of patients who had disclosed their symptoms to someone. Table 2 shows to whom female and male patients with a partner disclosed their symptoms.

The median PD was 12 days (range: 0–843 days). The 25th and 75th percentiles of PD were 1 day and 55 days, respectively. Hence, a short PD was ⩽1 day, a medium PD was 2–55 days and a long PD was >55 days. Mean scores and standard deviations of the four social support subscales in the three PD groups are shown in Table 3 .

The unadjusted relationships between the length of PD and each of the social support measures are shown in Table 4 . In female patients, no differences in social support subscales and covariates were observed between patients with a short and a medium PD. When female patients with a long PD were compared with female patients with a medium delay, Partner Support, Other Avoidance and Other Support were found to be associated with PD. Hence, increasing levels of Partner Support and Other Support significantly reduced the likelihood of having a long PD, whereas increasing levels of Other Avoidance significantly increased the likelihood of having a long PD in female cancer patients. Finally, the univariate analyses revealed that disclosure of symptoms significantly reduced the likelihood of having a long PD in female patients. The results of adjusting for the possible influence of age, educational level and family history of cancer are also shown in Table 4 together with all four social support measures. In female cancer patients, none of the support subscales and none of the covariates differed significantly between patients with a medium and a short PD. Levels of Other Avoidance and disclosure still differed between female patients with a medium and a long PD. Hence, female patients reporting high levels of Other Avoidance were more likely to have a long PD, and female patients who had disclosed their symptoms to others were less likely to have a long PD.

In male cancer patients, the factors found to be associated with PD in the univariate analyses were Other Support and relationship status. Thus, the likelihood of having a long PD increased significantly with decreasing levels of Other Support. This association was not maintained in the multivariate analysis. Male patients in a relationship were less likely to have a long PD. In the adjusted analysis, Partner Support was the only factor significantly associated with PD in male cancer patients. Hence, increasing levels of Partner Support significantly reduced the likelihood of having a long PD.

## Discussion

We found that social support was associated with the length of PD in patients with various cancer diseases. Results of univariate analyses revealed that female patients with a long PD reported less Partner and Other Support and more Other Avoidance than female patients with a medium PD. Moreover, female patients with a long PD were more likely not to have disclosed their symptoms to someone close to them. In male patients, being in a relationship reduced the risk of a long PD in the univariate analyses. One possible explanation for the Other Support and Other Avoidance subscales being significantly associated with PD in female patients but not with PD in male patients could be that more female patients than male patients were single. Thus, more female patients than male patients had to rely on social support from other sources than a partner.

In the multivariate analysis, Other Avoidance and disclosure of symptoms remained significantly associated with the length of PD in female cancer patients. In the multivariate analysis where relationship status was omitted, as only patients in a relationship were included in the analysis, the results revealed that male patients with a long PD reported less Partner Support than male patients with a medium PD.

The patterns of use of the social network observed in this study correspond to results of previous studies. Thus, we found that PD in female patients was influenced by both partner and Other Support and that Other Avoidance, in the multivariate analysis, was a more important factor for the length of PD than social support from the partner. In male patients, being in a relationship and receiving support from the partner were the two most important factors for reducing the length of PD. In agreement with these findings, previous studies have suggested that women have larger networks and are more likely to rely on friends and children for emotional support than men who are more likely to rely exclusively on their partners (Antonucci and Akiyama, 1987; Umberson, 1992; Ye *et al*, 2009). Moreover, one study found that men experienced greater distress when exposed to social constraints from their spouse than did women (Zakowski *et al*, 2003).

The results of this study showed that female cancer patients reported more Partner Avoidance than male patients who, on the contrary, reported more Partner Support than female patients. The higher levels of perceived Partner Support observed in male patients correspond to previous results (Kristofferzon *et al*, 2003; Goldzweig *et al*, 2009). We are not able to explain why female patients reported more Partner Avoidance than male patients, but in accordance with our results, it has previously been shown that women with a serious disease such as cancer receive less support from their partners than men with a serious disease do (Kristofferzon *et al*, 2003; Luszczynska *et al*, 2007). Recently, it has been suggested that most husbands and wives are equally skilled at providing support, but that wives are more responsive to their partners’ changing needs over time (Neff and Karney, 2005). On the basis of this, it could be anticipated that women are more sensitive to possible incongruity between the support that is needed and the support that is provided and, therefore, report more avoidant behaviour from the partner than men do.

Interestingly, in female patients disclosure of symptoms to someone was a more important factor for reducing the length of PD than being in a relationship. In agreement with this finding, one study of breast cancer patients showed that patients who did not disclose their symptoms to someone close to them were more likely to delay medical help-seeking (Burgess *et al*, 1998). In this study, nearly 60% of the female patients who were in a relationship had disclosed their symptoms to both the partner and other members of the social network. Approximately 14% of the female patients in a relationship had not disclosed their symptoms to anyone, and 3% of the female patients in a relationship had disclosed their symptoms to someone in the social network, but not to the partner. Further studies are needed in order to examine what these patterns of disclosure reflect, and whether the group of female patients in a relationship who do not disclose their symptoms to their partners is a group with special needs with regard to reducing the length of PD. According to the interpersonal process model of intimacy, self-disclosure is a key concept and is assumed to be a prerequisite of supportive relationships (Manne *et al*, 2004a, 2004b, 2010; Manne and Badr, 2009). By not disclosing the symptom to the partner, the individual who experiences a symptom obviously prevents himself from being encouraged to talk to the GP. Results from previous qualitative (Zola, 1973; de Nooijer *et al*, 2001; Scott *et al*, 2006, 2009) and quantitative studies (Eriksson *et al*, 2004) have revealed that the partner's worries are important triggers of consultations with the GP. The decision of the individual experiencing a symptom not to disclose the illness-related worries to the healthy partner might reflect thoughtfulness, but the lack of self-disclosure may also reflect low quality of the marital relationship and lack of relationship intimacy (Manne *et al*, 2004a; Badr and Taylor, 2006). Studies have shown that patients and their spouses in general find it difficult to talk about health-related worries, and that mutual social constraints are possibly greater in couples experiencing a relationship crisis (Badr and Taylor, 2006). Moreover, when a couple experiences distress, medical help-seeking may be impeded as actions aimed at making the relationship work are the number one priority (Scott *et al*, 2006). Unfortunately, the quality of the marital relationships was not addressed in this study. This issue and its influence on PD have to be investigated further in future studies.

The finding of this study suggesting that men rely on the partner only when discovering a symptom could indicate that singlehood has detrimental consequences to men, in particular concerning the health status. Results have shown that beginning a conversation about symptoms or health issues is in contrast to the images of masculinity of many men (Gascoigne *et al*, 1999) and, therefore, it may be a difficult task to alter the observed patterns of behaviour when men are confronted with a health threat. Insofar that the patterns of behaviour are difficult to change, the results of this study are suggestive that cancer awareness campaigns should be designed differently when targeted men or women. Women may benefit from being motivated to discuss health issues with adult children or friends, whereas awareness campaigns aimed at improving partnered men's health should motivate men to discuss health issues with their partner or – as a more radical suggestion – should teach women how to be attentive to the health of their husbands. Further research is needed in order to clarify how to address the needs of single men.

Instead of using the 25th and 75th percentiles for categorising PD, we could have used the conventional cut-off on 3 months. However, this cut-off, introduced by Pack and Gallo (1938), has been criticised for not being based on evidence (Scott and Walter, 2010). Alternatively, cut-offs considered to be clinically meaningful could have been used, but as patients with various forms of cancers with different clinical guidelines for seeking medical attention were included in this study, the use of clinically meaningful cut-offs did not appear appropriate. Optimally, the time taken to seek medical help is treated as a continuous variable, but as a consequence of skewed data it is recommended that the median are presented to prevent extreme cases (Scott and Walter, 2010). We followed this recommendation and widened it by using the 25th and 75th percentiles, which allowed us to examine whether those patients with an extremely short delay differed from patients with a medium PD.

Some limitations of this study should be noted. First, 260 of the questionnaire responses were excluded as the patients did not provide sufficient data concerning the length of PD, and further 82 patients were excluded as a consequence of missing responses on the social support subscales. The excluded patients were older than the included patients. There is no conclusive evidence that age is associated with the length of PD (Macleod *et al*, 2009), but as age is likely to be associated with relationship status and the size of the social network, the exclusion of patients may have caused selection bias of unknown size and direction. Second, as a consequence of the cross-sectional study design, direction of causality cannot be determined. Third, the study is retrospective and the risk of possible recall bias cannot be excluded. However, the risk of reduced reliability and validity are well-known methodological limitations in studies of PD (Andersen *et al*, 2009). Fourth, this study revealed differences in the levels of social support between patients with a medium and a long PD. The multivariate analyses, however, revealed no differences between patients with a medium and a short PD. As all other groups were compared with the group of patients with a medium PD, the results did not show whether patients with a long delay differed from patients with a short delay. As both a very long and a very short PD may reflect inappropriate illness behaviour, future studies should examine the differences between these groups. Fifth, the multivariate analysis was based on patients in a relationship only, and the number of single patients was rather low reducing the statistical power below acceptable levels when performing analyses with this group of patients. Thus, further studies are needed in order to examine whether patients without a partner differ in how social support affects them compared with patients with a partner.

In conclusion, the results of this study revealed that less Partner Support, less Other Support, more Other Avoidance and non-disclosure of the symptom(s) were associated with a long PD in female cancer patients. In the multivariate analysis, including only patients in a relationship, non-disclosure of symptom(s) and Other Avoidance remained to be significantly associated with a long PD in female cancer patients. In male cancer patients, being in a relationship and Partner Support were the factors most strongly associated with decreased risk of a long PD. The results of this study suggest that female and male cancer patients differ in their ways of using the social network when exposed to a health threat, and these gender differences may explain some of the mixed findings in previous studies examining the relationship between social support and PD.

## Figures and Tables

**Table 1:** Sociodemographic characteristics and perceived reactions of the partner and others

**Characteristic**	**Women (*n*=487)**	**Men (*n*=423)**	***P*-value**
Continuous variables	Mean (s.d.)	Mean (s.d.)	
Age (years)	59.8 (14.7)	64.2 (12.8)	<0.001
			
*Social support subscales*			
Partner Avoidance	0.68 (0.59)	0.48 (0.45)	<0.001
Partner Support	1.47 (0.71)	1.67 (0.72)	<0.001
Other Avoidance	0.57 (0.61)	0.51 (0.53)	0.13
Other Support	1.35 (0.70)	1.04 (0.69)	<0.001
			
**Categorical variables**	***N* (%)**	***N* (%)**	
*Educational level*			
Lower secondary or none	126 (25.9)	86 (20.3)	0.10
Upper secondary	198 (40.7)	195 (46.1)	
Tertiary	149 (30.6)	131 (31.0)	
Missing information	14 (2.9)	11 (2.6)	
			
*Relationship status*			
In a relationship	337 (69.2)	361 (85.3)	<0.001
Single	150 (30.8)	62 (14.7)	
			
*Many relatives with cancer*			
No	301 (61.8)	279 (66.0)	0.33
Yes	172 (35.3)	139 (32.9)	
Missing information	14 (2.9)	5 (1.2)	
			
*Disclosure*			
No	83 (17.0)	76 (18.0)	0.67
Yes	393 (80.7)	334 (79.0)	
Missing information	11 (2.3)	13 (3.1)	

**Table 2:** Description of to whom female (*N*=337) and male patients (*N*=361) with a partner disclosed their symptoms

**Symptoms disclosed to:**	**Female patients in a relationship, *N* (%)**	**Male patients in a relationship, *N* (%)**
The partner only	79 (23.4)	118 (32.7)
Other members of the network only	11 (3.3)	0 (0.0)
Both the partner and other members of the network	198 (58.8)	179 (49.6)
Neither the partner nor other members of the network	45 (13.4)	54 (15.0)
Missing information	4 (1.2)	10 (2.3)

**Table 3:** Mean item scores and s.d. on the four social support subscales in patient delay groups

	**Patient delay groups**		
	**Short patient delay mean item score (s.d.)**	**Medium patient delay mean item score (s.d.)**	**Long patient delay mean item score (s.d.)**
*Female patients*			
Partner support	1.62 (0.70)	1.52 (0.70)	1.23 (0.68)
Partner avoidance	0.60 (0.55)	0.66 (0.57)	0.79 (0.67)
Other support	1.41 (0.71)	1.40 (0.66)	1.18 (0.74)
Other avoidance	0.48 (0.59)	0.52 (0.54)	0.76 (0.70)
			
*Male patients*			
Partner support	1.78 (0.66)	1.67 (0.74)	1.54 (0.43)
Partner avoidance	0.44 (0.44)	0.53 (0.46)	0.42 (0.43)
Other support	1.19 (0.71)	0.94 (0.72)	1.03 (0.60)
Other avoidance	0.48 (0.53)	0.54 (0.55)	0.49 (0.51)

**Table 4:** Results of univariate and multivariate multinomial logistic models in female and male cancer patients

	**Dependent variable: length of patient delay**											
	**Short: ⩽1 day**						**Long: >55 days**					
**Medium: 2–55 days**	**Unadjusted**			**Adjusted^b^**			**Unadjusted**			**Adjusted^b^**		
**(Reference)^a^**	**RRR**	**95% CI^c^**	***P*-value**	**RRR**	**95% CI^c^**	***P*-value**	**RRR**	**95% CI^c^**	***P*-value**	**RRR**	**95% CI^c^**	***P*-value**
*Female cancer patients (N=487)*												
Partner avoidance	0.83	0.51–1.35	0.45	0.78	0.39–1.54	0.47	1.43	0.94–2.18	0.10	0.73	0.38–1.38	0.33
Partner support	1.22	0.83–1.79	0.32	1.16	0.68–1.98	0.57	0.54	0.37–0.80	0.002	0.62	0.36–1.07	0.09
Other avoidance	0.88	0.59–1.32	0.55	1.31	0.69–2.48	0.41	1.81	1.25–2.60	0.002	2.54	1.40–4.59	0.002
Other support	1.02	0.75–1.41	0.89	0.99	0.57–1.73	0.97	0.63	0.45–0.89	0.009	0.76	0.43–1.34	0.35
Age	1.00	0.99–1.02	0.63	1.00	0.98–1.02	0.94	0.99	0.97–1.00	0.08	1.00	0.98–1.02	0.92
												
*Educational level*												
Lower secondary^d^	0.59	0.38–0.93	0.02	0.71	0.32–1.55	0.39	0.61	0.38–0.96	0.03	0.79	0.36–1.74	0.56
Tertiary^e^	1.24	0.78–1.97	0.36	0.87	0.37–2.07	0.75	1.17	0.72–1.89	0.52	1.30	0.55–3.08	0.56
												
*Relationship status*												
Single (ref)	1.00						1.00					
In a relationship	0.65	0.42–1.01	0.06	Omitted^f^			1.59	0.94–2.69	0.08	Omitted^f^		
												
*Relatives with cancer*												
Few (ref)	1.00			1.00			1.00			1.00		
Many	1.15	0.73–1.80	0.55	1.56	0.84–2.92	0.16	1.17	0.74–1.86	0.51	1.10	0.58–2.08	0.77
												
*Disclosure of symptoms*												
No disclosure (ref)	1.00			1.00						1.00		
Disclosure	0.69	0.38–1.26	0.23	0.68	0.23–2.03	0.49	0.35	0.20–0.62	<0.001	0.25	0.10–0.63	0.003
												
*Male cancer patients (N=423)*												
Partner avoidance	0.63	0.36–1.11	0.11	0.87	0.45–1.67	0.67	0.57	0.32–1.04	0.07	0.73	0.37–1.47	0.38
Partner support	1.24	0.87–1.77	0.24	0.95	0.61–1.47	0.81	0.77	0.54–1.10	0.15	0.54	0.34–0.85	0.008
Other avoidance	0.81	0.52–1.26	0.35	0.71	0.41–1.24	0.23	0.83	0.53–1.32	0.44	0.85	0.49–1.47	0.56
Other support	1.68	1.19–2.38	0.003	1.47	0.95–2.27	0.09	1.22	0.85–1.74	0.27	1.25	0.78–2.02	0.35
Age	0.99	0.97–1.01	0.33	0.99	0.97–1.01	0.25	0.98	0.96–1.00	0.05	0.98	0.96–1.00	0.08
												
*Educational level*												
Lower secondary^d^	1.18	0.75–1.87	0.48	1.01	0.47–2.15	0.99	0.91	0.57–1.47	0.71	0.99	0.44–2.24	0.99
Tertiary^e^	0.69	0.41–1.14	0.15	0.86	0.37–2.01	0.73	1.01	0.61–1.67	0.97	1.19	0.50–2.87	0.70
												
*Relationship status*												
Single (ref)	1.00						1.00					
In a relationship	0.54	0.28–1.04	0.07	Omitted^f^			0.51	0.26–1.00	0.05	Omitted^f^		
												
*Relatives with cancer*												
Few (ref)	1.00			1.00			1.00			1.00		
Many	1.14	0.71–1.85	0.58	1.20	0.67–2.16	0.55	0.82	0.49–1.36	0.44	1.18	0.63–2.19	0.61
												
*Disclosure of symptoms*												
No disclosure (ref)	1.00			1.00			1.00			1.00		
Disclosure	1.57	0.82–2.98	0.17	1.27	0.55–2.89	0.58	0.82	0.46–1.46	0.50	0.83	0.39–1.81	0.65

Abbreviation: RRR=relative risk ratio.

a Reference refers to the group that all other groups are compared with in the multinomial model.

b Only patients with a partner were included in the multivariate multinomial regression analyses (*N*=337 and 361 for females and males, respectively).

c 95% Confidence interval for relative risk ratio.

d Patients with lower secondary education or no education were compared with patients with upper secondary education and patients with tertiary education combined (reference).

e Patients with tertiary education were compared with patients with lower secondary or no education and patients with upper secondary education combined (reference).

f The variable ‘Relationship status’ was omitted from the multivariate multinomial regression analyses as these analyses only included patients with a partner.

## APPENDIX

Partner Support (Cronbach's *α*=0.79)

My partner asked about my symptoms

My partner took the initiative to talk about my concerns

My partner advised me to talk to my physician

My partner tried to calm me

My partner talked directly about cancer

Partner Avoidance (Cronbach's *α*=0.71)

My partner minimised my concerns

My partner pretended nothing had happened

My partner avoided talking about cancer

My partner hid his/her concerns

My partner was not worried

Other Support (Cronbach's *α*=0.79)

Other asked about my symptoms

Other took the initiative to talk about my concerns

Other advised me to talk to my physician

Other tried to calm me

Other talked directly about cancer

Other Avoidance (Cronbach's *α*=0.81)

Other minimised my concerns

Other pretended nothing had happened

Other avoided talking about cancer

Other hid his/her concerns

Other was not worried
